# A Mamba U-Net Model for Reconstruction of Extremely Dark RGGB Images

**DOI:** 10.3390/s25082464

**Published:** 2025-04-14

**Authors:** Yiyao Huang, Xiaobao Zhu, Fenglian Yuan, Jing Shi, Kintak U, Junshuo Qin, Xiangjie Kong, Yiran Peng

**Affiliations:** 1 Faculty of Innovation Engineering, Macau University of Science and Technology, Macau 999078, China; 2 School of Information Engineering, Nanchang Hangkong University, Nanchang 330063, China; 3 Department of Mechanical & Materials Engineering, University of Cincinnati, Cincinnati, OH 999039, USA

**Keywords:** extreme dark light, image enhancement, Mamba U-Net, RGGB images

## Abstract

Currently, most images captured by high-pixel devices such as mobile phones, camcorders, and drones are in RGGB format. However, image quality in extremely dark scenes often needs improvement. Traditional methods for processing these dark RGGB images typically rely on end-to-end U-Net networks and their enhancement techniques, which require substantial resources and processing time. To tackle this issue, we first converted RGGB images into RGB three-channel images by subtracting the black level and applying linear interpolation. During the training stage, we leveraged the computational efficiency of the state-space model (SSM) and developed a Mamba U-Net end-to-end model to enhance the restoration of extremely dark RGGB images. We utilized the see-in-the-dark (SID) dataset for training, assessing the effectiveness of our approach. Experimental results indicate that our method significantly reduces resource consumption compared to existing single-step training and prior multi-step training techniques, while achieving improved peak signal-to-noise ratio (PSNR) and structural similarity (SSIM) outcomes.

## 1. Introduction

Images in extremely dark light often have low brightness, poor contrast, a narrow grayscale range, and color distortion. These issues can significantly impact how the human eye perceives the images and can also limit the performance of computer vision systems. The recovery of images captured in extreme dark-light conditions represents a significant challenge for digital image processing. This work necessitates implementing image processing techniques to enhance the quality of images captured in exceedingly dark-scene conditions. RGGB images are predominantly observed in mobile devices, including cell phones, cameras, drones, and other devices that generate high-resolution capture conditions. Images captured at night or in extreme darkness frequently fail to accurately represent the content, negatively impacting the image quality and its subsequent usability in imaging, identification, and detection work or agricultural and industrial production.

**Figure 1:**
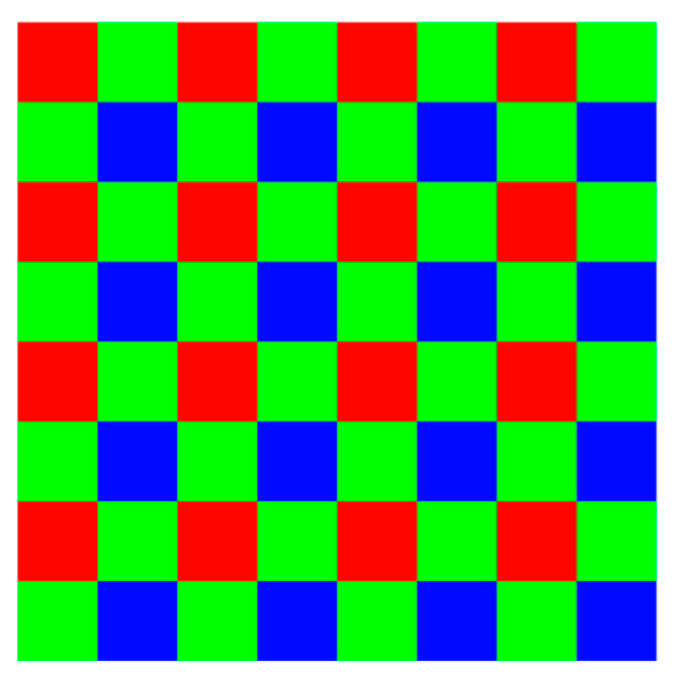
Structure of the RGGB image format.

In digital image processing, images captured by mobile cameras in RGGB format are vital for research datasets. This format, as depicted in Figure 1, with its unique arrangement of color filters—two green filters, one red filter, and one blue filter—poses a distinct challenge during the preprocessing stage of image analysis, particularly in training neural networks for diverse applications.

**Figure 2:**
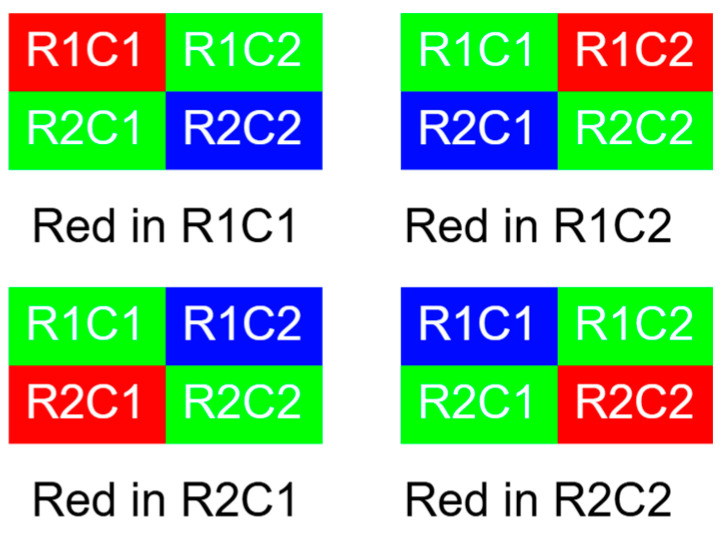
Different arrangements of red components in RGGB images.

The design and structure of raw-format images are closely related to the physiological characteristics of human vision, especially the sensitivity to the green spectrum. This sensitivity is attributed to the higher density of green-sensitive cones in the human retina, which significantly affects our perception of color and detail in the visual field. Therefore, to mimic this natural tendency and improve the fidelity of digital images, each 2 × 2 matrix in a typical raw format image contains a specific arrangement of color components: two dedicated to green (G), one to red (R), and one to blue (B). This configuration, shown in Figure 2, is not arbitrary but rather a deliberate attempt to optimize image quality by exploiting the inherent color sensitivity of the human eye. Therefore, most mobile cameras use the RGGB arrangement to enhance image quality.

The Mamba model [1] is based on the selective state space model (SSM) [2]. SSM has demonstrated excellent performance on long sequences. Formally, SSM utilizes the following ordinary differential equation (ODE) to model the input data:

(1) $$x^{\prime }(t)=Ax(t)+Bu(t),$$

(2) y(t)=Cx(t),

where x(t) is the state vector, u(t) is the input signal, and **A**, **B**, **C** are learnable matrices. SSM is designed to improve the ability to model long sequences while maintaining computational efficiency. Mamba combines the advantages of the state space model and overcomes the complexity and memory consumption issues of the transformer model when processing long sequences. Mamba replaces traditional attention with a state space model inspired by control theory mechanisms, using a multilayer perceptual machine (MLP) for computation. The selective state space mechanism at the core of Mamba allows the model to selectively propagate or forget information based on the current token along the sequence length dimension, thereby improving the effectiveness of lengthy sequence modeling. The Mamba model ($O_{\left(n\right)}$) is significantly better than the transformer model ($O_{\left(n^{2}\right)}$) [1,2] in terms of time complexity, especially for processing million-level token sequences. Additionally, the Mamba model introduces a selectivity mechanism that allows it to adjust SSM parameters dynamically according to the input, filtering out irrelevant information and retaining important data. Moreover, the Mamba model adopts scanning instead of the convolutional computation method, effectively improving speed.

Two complementary metrics are adopted to evaluate the performance of low-light image restoration quantitatively: peak signal-to-noise ratio (PSNR) and structural similarity index (SSIM) [3]. PSNR measures pixel-level fidelity by computing the logarithmic ratio between the maximum possible signal power and the mean squared error (MSE) between the reference and reconstructed images, defined as follows:

(3) $$PSNR=10\cdot {log}_{10}(\frac{{MAX}^{2}}{MSE}),$$

where MAX is the maximum pixel value (e.g., 255 for 8-bit images). While PSNR provides a straightforward assessment of noise suppression, it may fail to align with human perception due to its ignorance of structural correlations. SSIM addresses this limitation by evaluating the similarity in luminance, contrast, and structure between images, expressed as follows:

(4) $$SSIM(x,y)=(\frac{\left(2\mu_{x}\mu_{y}+C_{1})(2\mu_{xy}+C_{2}\right)}{\left(\mu_{x}^{2}+\mu_{y}^{2}+C_{1})(\sigma_{x}^{2}+\sigma_{y}^{2}+C_{2}\right)}),$$

where μ, σ, and $\mu_{xy}$ denote local means, variances, and covariance, respectively. SSIM ranges from 0 to 1, with higher values indicating better perceptual quality. The joint use of PSNR and SSIM allows a balanced numerical accuracy and perceptual fidelity assessment.

The U-Net architecture [4] is a widely adopted encoder–decoder framework that performs well in tasks that require accurate spatial detail recovery. Its encoder progressively extracts hierarchical features through downsampling, while the decoder reconstructs high-resolution outputs through transposed convolutions. The skip connections between encoder and decoder layers fuse multi-scale information and preserve fine textures, which is crucial for recovering dark images with low signal-to-noise ratio. Although CNNs and transformers are common in image restoration, they face limitations in extreme low-light conditions. For example, the Sharp U-Net [5] designed for biomedical segmentation reduces computational cost by 40% using deep convolutions, but has difficulty modeling global illumination due to its local operations. Similarly, the transformer-based U-Net variant [6] achieves global context modeling through a self-attention mechanism, but its quadratic complexity is too high for high-resolution images.

In contrast, the SSM-based Mamba [1] addresses these challenges with linear time complexity (O(n)) and selective state propagation. Mamba is integrated into the U-Net encoder to dynamically filter noise and prioritize lighting-critical areas by serializing spatial features into a one-dimensional sequence. This design avoids the parameter redundancy of CNN and the computational overhead of transformer. Combined with skip connections for local detail recovery, our framework achieves efficient reconstruction while improving fidelity.

Previous studies mainly used CNN- and transformer-based methods, which require a lot of resources and long training time. Extremely dark images require end-to-end training to achieve the best results, and existing multi-stage methods require 7.7 M parameters and 48.5 G FLOPs. By replacing CNN with Mamba modules, our single-step training network reduces the number of parameters by 65% and FLOPs by 56%, while maintaining highly competitive performance. The proposed pipeline first converts the RGGB input to RGB through linear interpolation, compressing the data to 5% of the RAW size without color cast, then restores details through Mamba U-Net, and finally applies a lightweight gray-world white balance module to adaptively neutralize residual color bias. This holistic approach balances efficiency, fidelity, and practicality, providing a powerful solution for resource-constrained low-light imaging scenarios.

## 2. Related Works

Much research has been dedicated to enhancing images captured in low-light and extremely dark environments. However, there are notable limitations concerning the datasets utilized for training: most datasets categorized as low-light imaging primarily consist of photos taken during daylight. This results in a critical shortage of effective training materials for models intended to operate under extremely dark conditions. Our approach utilizes the SID dataset, which comprises extremely dark images for training purposes.

**Figure 3:**
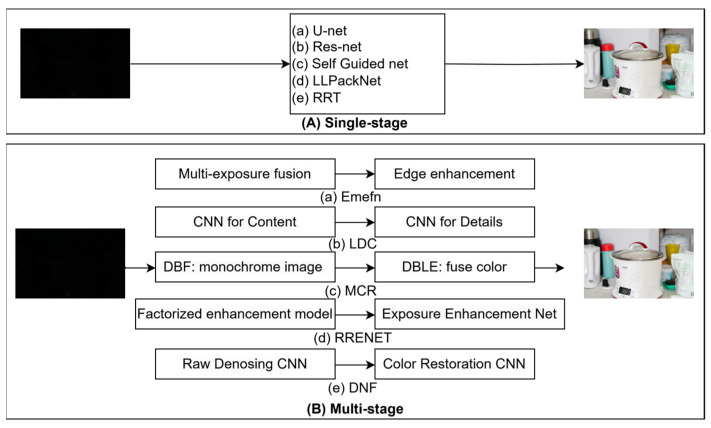
Processing extremely dark RGGB images typically involves either single-step or multi-step methods. Each box in the diagram represents an encoder–decoder framework.

As illustrated in Figure 3, learning-based methods can be categorized into two primary forms: single-step and multi-step approaches. Each method typically involves end-to-end training. However, single-step training often struggles to address issues such as underexposure and color cast effectively in a single attempt. Consequently, many studies have shifted towards multi-step training to tackle these challenges. Unfortunately, this approach frequently results in significant resource consumption and prolonged processing times. Our research focuses on achieving a balance between performance and resource consumption to attain a more optimal outcome.

Below is a summary of the current research in this domain.

### 2.1. Traditional Algorithms

The traditional methods for processing low-light images include grayscale transformation, histogram equalization, Retinex, frequency domain, and more [7]. These methods can be further classified into subclasses based on underlying principles. Each method is detailed below.

The grayscale transformation method is a spatial domain image enhancement technique where the grayscale value of each pixel is converted to other grayscale values using a mathematical function [8]. The grayscale transformation includes linear and nonlinear transformations. Linear transformation [9] adjusts the gray value directly using a linear function, while nonlinear transformation uses a nonlinear function to transform the gray value of the image. Nonlinear transforms include logarithmic, gamma, and other improved functions [10,11] to better handle images under dark illumination conditions. Logarithmic transformation extends the dark pixel values, while gamma transformation improves the sensitivity to dark details.

Histogram equalization [12] improves the visual effect by adjusting the grayscale histogram of the image to make the distribution of pixel values more uniform. First, the input image’s gray-level histogram and cumulative distribution function (CDF) are computed. Then, the gray level values of the original image are converted to a new uniform distribution by a mapping function. In this way, the hidden details in the dark regions can reappear, and the visual effect of the input image can be effectively enhanced.

The Retinex method [13] is based on modeling color perception and color invariance of the human eye. It decomposes the image into reflection and illumination components to achieve color constancy. By estimating the illumination component, Retinex methods can eliminate the effect of uneven illumination and improve the visualization of the image. The main types include single scale Retinex (SSR) [14], multi-scale Retinex (MSR) [15], and multi-scale Retinex with color recovery (MSRCR) [16].

The frequency domain method [17] involves transforming an image from the spatial domain to the frequency domain using Fourier transform for filtering and enhancement. In the frequency domain, high-frequency components represent the image details and edge information, while low-frequency components correspond to overall contours and illumination changes. The main steps include Fourier transform, frequency-domain filtering (applying high-pass or low-pass filters), inverse Fourier transform, and finally, converting the processed spectrum back to the spatial domain to obtain an enhanced image.

### 2.2. Single-Stage Learning-Based Algorithms

For extremely dark RGGB images, a common solution is to train end-to-end pipeline networks, as described in more detail below:

The seeing in the dark (SID) [18] system utilizes a U-Net network for image processing to enhance performance in low-light conditions by learning contrast. The model takes a very dark image as input and produces an improved image. The seeing motion in the dark (SMID) [19] system incorporates a motion estimation framework based on an enhanced Res U-net. This framework leverages information from multiple frames to improve the accuracy and reliability of motion detection.

Based on the two datasets and their methods, recent studies focused on improving them. Cai et al. [20] used an enhanced U-Net architecture that combines recursive residual convolutional units (RRCUs) and dilation convolution to improve feature extraction and image reconstruction. Maharjan et al. (DID) [21] proposed a deep network method based on residual learning to enhance image denoising under very low light conditions. The method effectively captures complex features by learning the differences between the original and denoised images, thus effectively removing noise. Gu et al. (SGN) [22] employ a self-guided strategy to process the image by creating input variants with different spatial resolutions. This allows the network to learn and denoise at different levels, thereby improving denoising effectiveness and efficiency. Lamba et al. proposed two approaches to this problem. Lamba et al. (LLPackNet) [23] utilize an adaptive amplification module and introduce “Pack” and “UnPack” operations dependent on the input original image. This approach estimates the amplification factor directly from the input raw image without requiring the real exposure value, making it compatible with the pre-training model. Lamba et al. (RRT) [24] perform most of the processing in a high-scale space and attempt to skip the intermediate scale as much as possible. Combined with the pre-amplification module, it demonstrates good generalization ability and can combine speed, efficiency, and quality to promote application in real scenarios.

### 2.3. Multi-Stage Learning-Based Algorithms

Many studies have focused on improving individual end-to-end imaging through multiple steps to achieve higher-quality results. While these approaches often lead to better results, they require more resources and processing time. Below are descriptions of studies from recent years:

Zhu et al. (EEMEFN) [25] introduced the edge-enhanced multi-exposure fusion network, a two-stage approach. A multi-exposure fusion module addresses high contrast and color bias issues in the first stage. The second stage integrates an edge enhancement module to refine the initial image further using edge information. This allows for reconstructing high-quality images with sharp edges while minimizing pixel-by-pixel loss. Xu et al. (LDC) [26] proposed a frequency decomposition and enhancement model that includes an attention context encoding (ACE) module and a cross-domain transform (CDT) module. This model decomposes the image into low and high-frequency layers. The low-frequency layer recovers objects and colors, while the high-frequency layer enhances the details. Dong et al. (MCR) [27] suggested using an alternative sensor architecture to capture complete color information without relying on Bayer filters. Deep learning models are then employed to capture the full spectrum of the data and learn to enhance low-light images rather than relying on interpolated values from Bayer filters. Huang et al. (RRENet) [28] proposed sample-less domain adaptation methods that utilize existing source camera labeling data and a small number of labeling samples from the target camera to enhance the quality of target domain imaging in very low light conditions. This approach achieves enhancement performance similar to or better than that achieved by training a model using a large-scale labeled target camera dataset with only ten or fewer target camera labeling samples. Jin et al. (DNF) [29] introduced the factorized enhancement model (FEM) and the RAW guided exposure enhancement network (REENet). The FEM method breaks down the characteristics of the RAW image into measurable factors. Meanwhile, the REENet uses RAW images for guidance and does not rely on RAW images in the testing phase. This reduces modeling by projecting the sRGB image into the linear RAW domain and applying constraints in combination with the corresponding RAW image.

Our previous research [6] employed a two-stage transformer-based self-attention U-Net model alongside an enhanced exposure fusion HDR model to restore extremely dark images. The self-attention U-Net model is designed to eliminate the black level of the image and restore its color. The enhanced exposure fusion HDR model utilizes the principles of HDR image generation. Initially, an end-to-end model produces a series of exposure-enhanced images. After applying algorithmic screening, the most suitable images are fused together to create an 8-bit pseudo-HDR image as the final output. Previous research concentrated on maximizing image quality without considering performance consumption. In contrast, our current research aimed to find a balance between performance and resource consumption. We focus on reducing the consumption involved in training and generation while still ensuring a certain level of image quality.

### 2.4. Datasets Used in Literature

Some datasets are limited in their variation of scenes and conditions, containing only a single sample type. For instance, the RENOIR [30] and DND [31] datasets primarily consist of low-ISO and high-ISO images, lacking a wide range of real scene pairs. The LOL dataset [32] includes 485 pairs of low-light and normal-light images. However, its relatively small sample size restricts training effectiveness and the generalization ability of deep learning models. Similarly, the exclusive dark dataset [33] comprises only ten different types of low-light images, limiting its applicability in various environments. LSRW [34] contains 500 low-light/normal-light image pairs but does not cover extremely dark-light images.

In contrast, the SID [18] dataset comprises 5094 short-exposure extreme low-light RAW images and 424 corresponding long-exposure reference images featuring both indoor and outdoor scenes across a diverse array of low-light conditions suitable for model training. This dataset has been available since 2018, and numerous studies utilizing it have been published in recent years in prestigious journals [28] and at major computer vision conferences, such as CVPR [18,24,26,27,29] and ICCV [19,22]. For our study, we utilized the SID [18] Sony dataset as the training set and conducted our tests on this same dataset.

## 3. Methodology

### 3.1. Proposed Pipeline Structure

**Figure 4:**
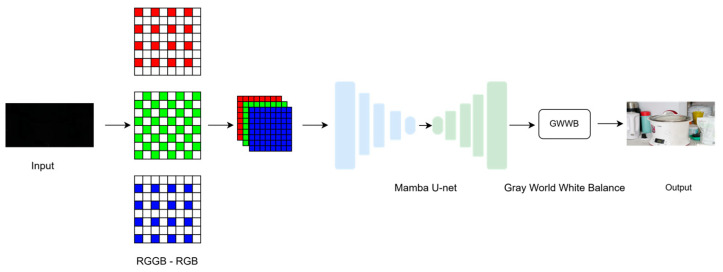
Schematic of the proposed pipeline consists of sequential steps for the RGGB to RGB algorithm and the Mamba U-Net network.

The image processing workflow, as depicted in Figure 4, starts with reducing the dimensionality of the input image. This involves converting each RGGB image into an RGB image using a bilinear interpolation technique [35]. The resulting standard RGB images are inputted into the Mamba U-net network to produce standard dynamic range (SDR) images without black level. This workflow ensures that image details are preserved in various lighting conditions and enhances the overall visual quality, providing a more vivid and dynamic viewing experience.

### 3.2. Image Preprocessing: RGGB to RGB

The specific structure of RGGB images necessitates a conversion process to transform these images into the more universally applicable RGB format. This preprocessing step is pivotal in enhancing the efficiency of the trained network. Normalizing the dataset by converting RGGB images to RGB format simplifies the training process and enhances training efficiency. This normalization not only streamlines the network structure but also significantly reduces the computational load, thereby optimizing the overall performance of the image analysis system.

We adopt bilinear interpolation for RGGB-to-RGB conversion due to its optimal balance between efficiency and fidelity in extreme dark-light conditions. Unlike training-dependent methods such as CNN or transformer-based demosaicing, bilinear interpolation requires no additional training, operates in linear time, and seamlessly integrates with imaging pipelines. Experimental results demonstrate its near-ideal performance while compressing file sizes to 5% of the original RAW data. The process of bilinear interpolation involves several steps:

The red and blue pixels already have their respective color components, so there is no need to interpolate them. The missing green component can be calculated by taking the average of two neighboring green pixels horizontally and vertically. The average of the corresponding color pixels in the four corners is taken for the blue component in the red pixel and the missing red component in the blue pixel. For green pixels, the green component is known. For a green pixel in a row of red pixels, the missing blue component can be calculated by taking the average of the blue pixels on either side, and the missing red component can be calculated by taking the average of the red pixels above and below. The calculation is reversed for green pixels in a row of blue pixels. Interpolation calculation: For each pixel to be interpolated, find the neighboring pixels around it and calculate the weighted average. This step is the core of bilinear interpolation, where the color information around the pixel is considered, and the missing color components are calculated by linear interpolation.

**Figure 5:**
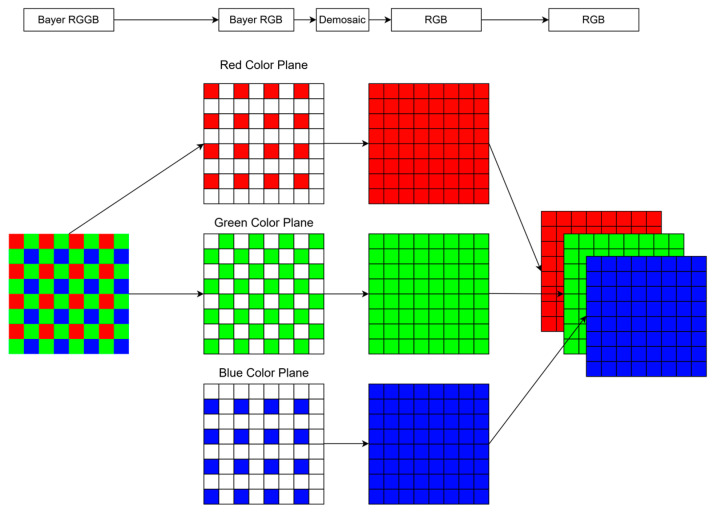
The RGGB to RGB interpolation process, which divides a mosaic image into three mosaic images, takes the white part of the image from the surrounding grid, calculates the weighted average, fills it out, and then synthesizes the three-color image.

In the RGGB image, the green color has the largest percentage because the human eye is more sensitive to green. Red and blue equally share the remaining portion. The interpolation process for obtaining a mosaic image involves dividing a mosaic image into three mosaic images. The white portion of the image is taken from the surrounding 3 × 3 squares, and a weighted average is calculated and filled. The three-color image is then synthesized. The dashed arrows in Figure 5 show the segmentation and synthesis of the RGGB, and the solid arrows show the result of the weighted average calculation followed by filling.

Neighboring green pixels refer to the green pixels that are adjacent to (i, j) in the horizontal (Δx = ±1, Δy = 0) or vertical (Δx = 0, Δy = ±1) direction in the RGGB pattern. The current pixel coordinates are (i, j), and the color component is red or blue $B_{\left(i,\;j\right)}$. Green component $G_{\left(i,\;j\right)}$ is calculated by the weighted average of adjacent green pixels:

(5) $$G_{i,j}=\frac{\sum_{\left(m,n\right)\in \{\left(0,-1\right),\left(0,1\right),\left(-1,0\right),(1,0)\}}G_{i+m,\;j+n}}{4}$$

Adjacent red pixels refer to red pixels located in the diagonal direction (Δx = ±1, Δy = ±1). Adjacent blue pixels refer to blue pixels located in the diagonal direction (Δx = ±1, Δy = ±1). The red component (i, j) at the blue pixel and the blue $\left(i,\;j\right)$ component at the red pixel are as follows:

(6) $$R_{i,j}=\frac{\sum_{\left(m,n\right)\in \{\left(-1,-1\right),\left(-1,1\right),\left(1,-1\right),(1,1)\}}R_{i+m,j+n}}{4}$$

(7) $$B_{i,j}=\frac{\sum_{\left(m,n\right)\in \{\left(-1,-1\right),\left(-1,1\right),\left(1,-1\right),(1,1)\}}B_{i+m,j+n}}{4}$$

The adjacent red pixel refers to the red pixel located in the vertical direction (Δy = ±1). The adjacent blue pixel refers to the blue pixel located in the vertical direction (Δy = ±1). The red component $\left(i,\;j\right)$ at the green pixel, and the blue component (i, j) at the green pixel are as follows:

(8) $$R_{i,j}=\frac{\sum_{\left(m,n\right)\in \left\{\left(0,-1\right),\left(0,1\right)\right\}\;or\;\{(-1,0),(1,0)\}}R_{i+m,j+n}}{2}$$

(9) $$B_{i,j}=\frac{\sum_{\left(m,n\right)\in \left\{\left(0,-1\right),\left(0,1\right)\right\}\;or\;\{(-1,0),(1,0)\}}B_{i+m,j+n}}{2}$$

In the process of image conversion from RGGB to RGB, the implementation of bilinear interpolation must strictly follow the distribution law of color-sensitive units in the RGGB format. The above formulas, $R_{\left(i,\;j\right)}$, $G_{\left(i,\;j\right)},$ and $B_{\left(i,\;j\right)}$ represent the green, red, and blue components at the pixel location (i, j), respectively. The summation is over the neighboring pixel offsets, corresponding to the same color component in the RGGB pattern. When completing the missing color component of a pixel, only the adjacent pixel values consistent with the target color channel (red, green, or blue) are selected for weighted summation, and the offset (Δx, Δy) needs to adapt to the spatial periodic distribution of color components in the RGGB pattern. For example, the blue component of a red pixel (located in odd rows and odd columns) needs to be interpolated through the blue pixel values of the four adjacent corners (Δx = ±1, Δy = ±1). In contrast, the red component of a green pixel needs to refer to the adjacent red pixels in the vertical direction (Δy = ±1). This method constrains the interpolation reference range to pixels of the same color. It utilizes the structured arrangement characteristics of the RGGB pattern to ensure the smoothness and spectral fidelity of color transitions while reducing computational complexity. It is the core optimization strategy of bilinear interpolation.

### 3.3. Mamba U-Net Network

**Figure 6:**
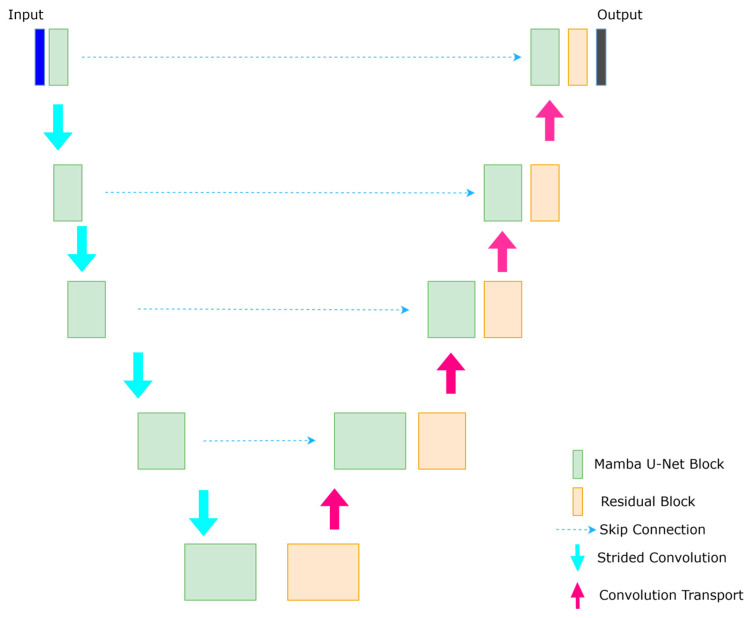
Mamba U-Net architecture consists of an encoder and decoder. The encoder has Mamba blocks, and the decoder has residual blocks and with skip connections.

Our Mamba U-Net architecture is based on the U-Net [35] structure, drawing inspiration from U-Net [35] and Swin-U-Net [36]. We incorporate elements from U-Mamba [37,38] and UVM-Net [39], known for their excellent performance in medical image segmentation and image defogging, respectively. In our architecture, the encoder comprises Mamba U-Net modules, while the decoder consists of residual blocks and transposed convolutions, which focus on capturing detailed local information and resolution recovery. Additionally, skip connections are used to link the hierarchical features of the encoder to the decoder, as illustrated in Figure 6.

The encoder consists of five hierarchical levels. The channel dimensions double at each level (3 → 6 → 12 → 24 → 48), while spatial resolution halves (512 × 512 → 256 × 256 → … → 16 × 16). The decoder symmetrically upsamples features via transposed convolutions (kernel = 3 × 3, stride = 2), with skip connections injecting multi-scale encoder features.

Mamba U-Net leverages an enhanced U-Net architecture to integrate global illumination modeling with local detail recovery through multi-scale feature interaction and dynamic parameter configuration in its encoder–decoder structure. We randomly crop a 512 × 512 patch for training and randomly augment the data using operations such as flipping and rotation.

The process begins with inputting a low-light RGB image with dimensions of (B, C, H, W) = (1, 3, 512, 512). The encoder comprises five levels of stacked U-Mamba blocks. At each level, downsampling is performed using strided convolution while the number of channels is doubled progressively. For instance, in the first level of the encoder, the input image undergoes processing through two residual blocks. Each of these residual blocks contains a 3 × 3 convolution layer (with a kernel size of 3 × 3, stride of 1, and padding of 1), instance normalization [40], and a leaky ReLU [41] activation function, all while maintaining the original spatial resolution of 512 × 512 and three channels. This step effectively extracts local noise patterns (such as sensor noise) and basic texture features.

The resulting feature map is then fed into the Mamba block, where the two-dimensional spatial features are flattened into a one-dimensional sequence, resulting in dimensions of (1, 3, 512 × 512) becoming (1, 3, 262,144). This sequence is then transformed into a serialized input of (1, 262,144, 3) through a transpose operation, making it suitable for the sequence modeling capabilities of the state-space model (SSM). The Mamba block emphasizes the dynamic selection of key illumination areas (such as the relationship between dark and highlight areas) through global dependency modeling with linear complexity. The block then outputs the reconstructed sequence features with dimensions (1, 262,144, 3).

The final step at this stage involves a strided convolution (kernel size of 3 × 3, stride of 2, padding of 1) to achieve spatial downsampling from 512 × 512 to 256 × 256 while expanding the number of channels from 3 to 6, forming the input for the second-level encoder (1, 6, 256, 256). This downsampling and channel expansion process continues through subsequent levels, increasing the number of channels to 12, 24, and 48 while reducing the spatial resolution to 16 × 16, thus constructing a multi-scale feature pyramid that captures the global illumination distribution.

The decoder features a symmetrical upsampling path that restores spatial resolution while reducing the number of channels through transposed convolution (kernel size of 3 × 3, stride of 2, padding of 1). For example, starting from the deepest feature representation (16 × 16 × 96), the transposed convolution increases the resolution to 32 × 32 while halving the number of channels to 48. This output is concatenated with features from the corresponding encoder level (32 × 32 × 48) via skip connections, resulting in a fused channel count of 96.

These concatenated features undergo processing through residual blocks [42] to mitigate artifacts introduced by upsampling and to refine local details. This progressive process restores spatial resolution from 32 × 32 to 512 × 512 while decreasing the number of channels from 48 to 3. The final output layer maps the channel count to the target dimension (RGB with three channels) using a 1 × 1 convolution and a sigmoid activation function [41] to create a normalized enhanced image with dimensions (1, 3, 512, 512).

The architecture’s effectiveness hinges on the Mamba block’s hierarchical design and the encoder’s residual block: the residual block preserves underlying details through local convolution. In contrast, the Mamba block captures global illumination relationships through serialization. The skip connections inject multi-scale global information (like dynamic range and color consistency) from the encoder into the decoder, guiding the local reconstruction process. Additionally, the network’s self-configuration mechanism, akin to that of nnU-Net, allows dynamic adjustments to the number of downsampling steps, channel cardinality, and residual blocks based on the input image’s resolution and task complexity. This flexibility ensures the model effectively balances efficiency and accuracy in addressing extremely low-light enhancement tasks.

### 3.4. Mamba U-Net Block

**Figure 7:**
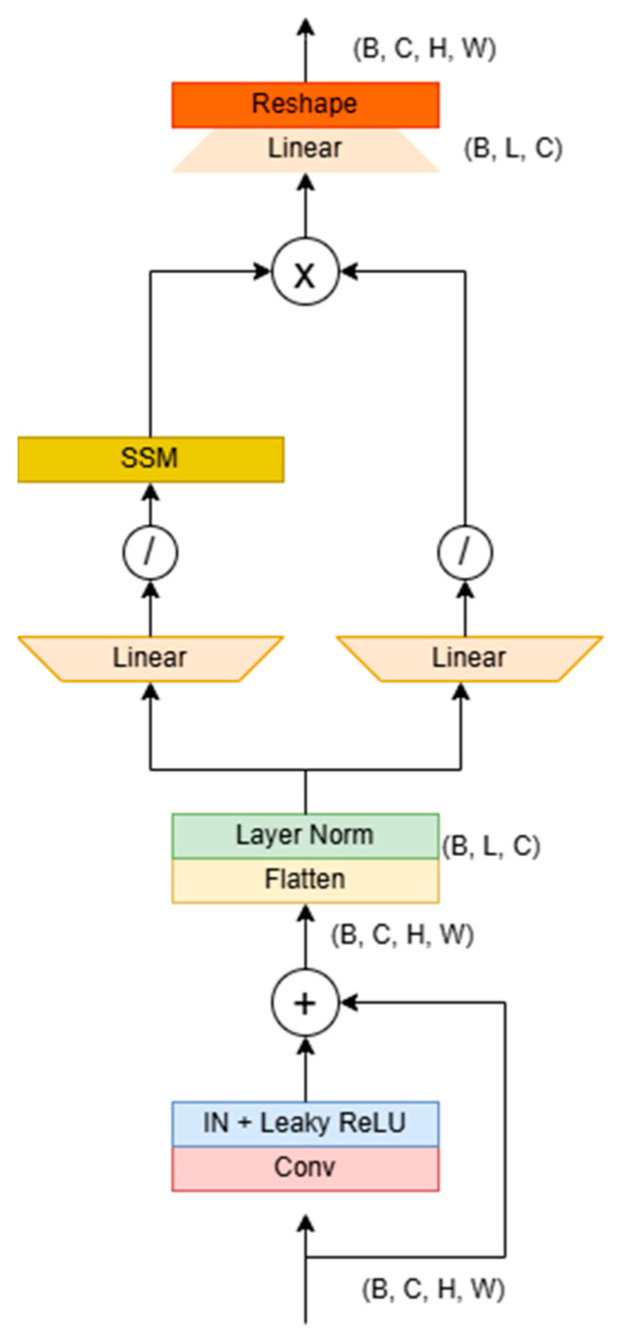
Mamba U-Net block details. The architecture combines two residual blocks and a Mamba U-Net block to obtain the reconstructed image using the Hadamard product through 1D SSM transformation and parallel branch processing.

The structure of the Mamba block is illustrated in Figure 7. Its primary purpose is to address the challenge of long-distance dependencies in images through serialization modeling. Initially, the module accepts local features extracted from the residual block [42], which typically have dimensions of (B, C, H, W). It then flattens the spatial dimensions (H×W) into a single sequence length L, resulting in dimensions of (B, C, L), and subsequently transposes this to (B, L, C) to meet the requirements of sequence modeling.

The module features a dual-branch architecture: the first branch expands the sequence length to 2L via a linear expansion layer, utilizing 1D convolution and the SiLU activation function [43] to capture local temporal patterns. It then models nonlinear relationships between global pixels, such as the brightness correlations found in the remote areas of dimly lit images through SSM. Meanwhile, the second branch also generates parallel features through linear expansion and activation, ultimately fusing the outputs of both branches via the Hadamard product before compressing the sequence length back to its original dimensions.

The fused features undergo transposition and reshaping to restore the spatial structure, resulting in a feature map that retains global dependency information. In extremely dim images, this architecture can dynamically identify key areas—enhancing brightness and suppressing noise in particularly dark regions—through serialized modeling while leveraging the linear complexity of SSM to associate pixels throughout the entire image efficiently. This approach effectively addresses the issues of uneven brightness or detail loss, commonly caused by local operations in traditional methods. Ultimately, the encoder–decoder framework facilitates the coordinated optimization of global illumination balance and local detail restoration.

### 3.5. Gray-World White-Balance Algorithm (GW-WB)

We implement a gray-world white-balance feedback mechanism to ensure that the image maintains a neutral color balance. In the output of our network, the average color values of the three RGB channels converge towards the same grayscale value, denoted as K. If the average of any channel deviates from the K value, the pixel values of that channel must be adjusted, with the correction depending on K. To prevent over-amplification, the gain coefficients ($K_{R},\;K_{G},\;K_{B}$) are clamped to the range of (0.8, 1.2), ensuring stable color correction across diverse scenes. As illustrated in Formula (10), the average of the three channels serves as the K value:

(10) $$K=\frac{R_{avg}+G_{avg}+B_{avg}}{3}$$

The gain of each channel relative to the K value is calculated as follows:

(11) $$K_{R}=\frac{K}{R_{avg}},K_{G}=\frac{K}{G_{avg}},\;K_{B}=\frac{K}{B_{avg}}$$

According to the Von Kries diagonal model, each pixel in the image is adjusted:

(12) $$R_{new}=R\times K_{R},G_{new}=G\times K_{G},\;B_{new}=B\times K_{B}$$

This module’s effectiveness relies entirely on the input image’s statistical characteristics. For instance, in low-light scenes that typically exhibit a blue cast, GW-WB will substantially enhance the gain of the red channel to counteract the cool tones. Conversely, the gain coefficient approaches 1 in scenes with balanced lighting to prevent unnecessary adjustments. This adaptability enables GW-WB to perform robust color correction without depending on predefined scene assumptions.

## 4. Experiments and Analysis

### 4.1. RGGB to RGB Experiments

**Figure 8:**
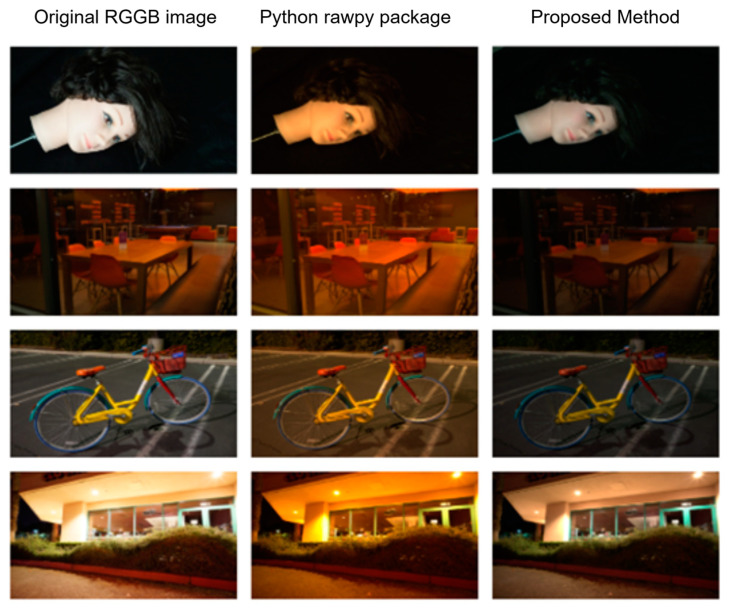
Comparison results of four distinct scenes are presented. The first column features the ground truth image from the dataset, the second column displays the processed image saved with the default parameters using the rawpy package in Python, and the third column showcases the results achieved through the proposed method.

We used interpolation techniques to convert RGGB images to RGB images. The original input image is a very dark scene, and it is not easy to compare the visual experience. Therefore, we used ground truth images for conversion experiments. The results are shown in Figure 8. The first column shows ground truth images from four different scenes, the second column shows the processed images saved with default parameters using the rawpy package in Python 3.11.2, and the third column shows the results obtained using the proposed method. From the results, the overall picture of the image processed by the traditional method has an obvious color cast, and the image is destroyed while reducing the dimension. Our method only loses some exposure from the visual point of view, and there is no color cast.

Table 1 presents four processed image sizes to showcase the outcomes of our dimensionality reduction techniques. We utilized the preprocessing method provided by the rawpy package in Python to read ARW images and subsequently convert them to PNG format in RGB for further processing. The compression results indicate that the original method significantly lags behind our approach in terms of effectiveness. This highlights the efficacy of our method in reducing data dimensionality and emphasizes its potential for preparing images for subsequent processing stages.

To quantify the conversion fidelity from RGGB to RGB, we adopt PSNR and SSIM as evaluation metrics. Our bilinear interpolation method achieves near-lossless performance on raw extremely dark-light inputs, effectively avoiding secondary artifacts like color casts, common in rawpy outputs or over-darkening. As shown in Table 2, it significantly outperforms the Python rawpy package. While learning-based methods might offer marginal theoretical gains, their prohibitive computational costs—10× higher GPU memory and 100× slower processing—render them impractical for real-world deployment. Given bilinear interpolation’s training-free nature, real-time capability, and near-optimal fidelity, it is the pragmatic and robust choice for extreme low-light imaging scenarios.

### 4.2. Comparison with Other State-of-the-Art Methods

We input the lowest condition with a minimum exposure time of 0.1 s to evaluate our method. The training process consists of 4000 epochs, employing the L1 loss function as the evaluation metric. During training, we maintain a batch size of 1, starting with an initial learning rate of 1 × 10^−4^, which is decreased to 1 × 10^−5^ at the 2000th epoch. We conducted the training on two NVIDIA T4 2*16 GB GPUs hosted on Tencent Cloud, with the total memory consumption being approximately 25 GB. This is significantly lower than the 48–52 GB typically required by other methods.

**Figure 9:**
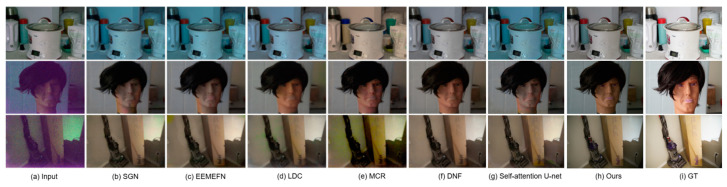
Comparison of the proposed Mamba U-Net approach with various learning-based approaches developed in recent years.

In our evaluation, we use the Sony dataset from SID [18] for training and comprehensively analyze the proposed method. Our comparisons include single-stage processes such as DID [21], SGN [22], LLPackNet [23], RRT [24], as well as multi-stage processes like EEMEFN [25], LDC [26], MCR [27], RRENet [28], DNF [29], and our previous work [6]. In previous studies (accepted and in publishing), we employed the self-attention U-Net network for recovery and have included it in our results for comparison. Figure 9 presents the results on the SID [18] dataset and illustrates how our method compares with others.

Figure 9 demonstrates that our method effectively restores extreme images, and notably, our single-step approach outperforms previous single-step methods. As illustrated in Table 3, our method achieves a PSNR improvement of 0.06 dB and an SSIM [3] enhancement of 0.003 compared to existing single-step processing techniques. While the data improvement may not appear substantial compared to other single-step and multi-step methods, our approach significantly reduces resource consumption during training. For instance, SID [18] exemplifies a single-step method, whereas our prior research, self-attention U-Net + HDR [6], represents a multi-step approach. We utilize the same cloud server for training, which features 2*16 GB+ of video memory, over 16 TFlops SP of computing power, a 16-core CPU, and 64 GB of RAM.

### 4.3. Computational Complexity Analysis and Resource Efficiency Verification

As demonstrated in Table 4, our method achieves a significant efficiency advantage over existing approaches. Compared with traditional CNN-based U-Net [18], Mamba U-Net significantly reduces the number of parameters and FLOPs, mainly due to its SSM-based architectural innovation and computational paradigm optimization. Traditional CNN-based U-Net relies on stacking multiple layers of local convolution kernels to gradually expand the receptive field, leading to significant parameter redundancy (such as repeated layer-by-layer calculation of 3 × 3 convolution kernels) and accumulating computational burden through multiple levels of downsampling and upsampling operations. In contrast, Mamba U-Net serializes images through SSM and uses the global dynamic system defined by the state matrix to capture long-range dependencies, eliminating the need for layer-by-layer parameter stacking associated with local convolution. The parameter count of SSM is only equal to the state dimension, and the parameters are shared in the sequence dimension, which greatly reduces the number of parameters. Additionally, the linear time complexity of SSM is significantly better than the square complexity of traditional convolution, and the selective scanning mechanism dynamically skips the calculation of redundant areas, further compressing FLOPs. Our experiments demonstrate that Mamba U-Net achieves superior reconstruction quality with only 2.3 M parameters and 20.9 G FLOPs, proving that its balanced design between efficiency and performance provides a feasible solution for resource-sensitive scenarios.

Despite these improvements, our single-step Mamba U-Net exhibits a slight performance gap compared to advanced multi-stage methods. Future work will focus on integrating lightweight post-processing modules to bridge this gap without compromising efficiency.

### 4.4. Ablation Studies

**Figure 10:**
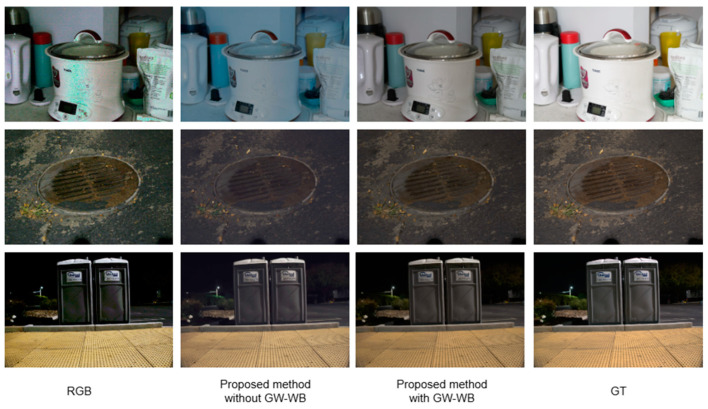
Comparison of standard U-Net and our method (with or without GW-WB).

To evaluate the effectiveness of Mamba U-Net in enhancing extremely dark-light images, we analyzed the performance differences between the conventional U-Net and Mamba U-Net under identical training conditions. Initially, the original RGGB array was transformed into an RGB image using bilinear interpolation, followed by end-to-end training conducted with both conventional U-Net [19] and Mamba U-Net. The results are presented in Figure 10. Further analysis of the scene indicates that the color correction capabilities of the GW-WB module are significantly influenced by the scene’s content. GW-WB can effectively correct the blue bias resulting from differences in dark-light quantum efficiency in scenes predominantly featuring a white background. However, the imaging results with GW-WB do not differ markedly from images processed without GW-WB in natural scenes.

**Figure 11:**
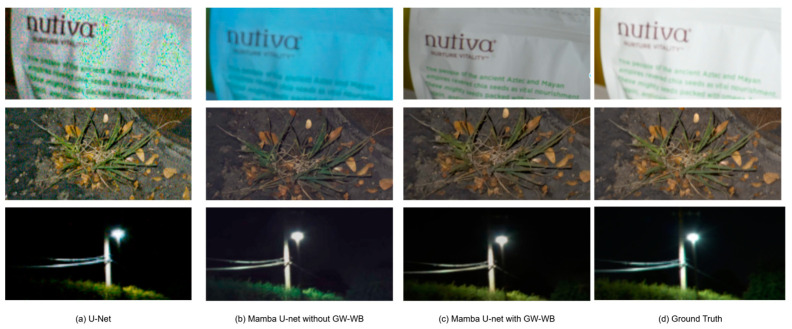
Comparison of enlarged image details between the standard U-Net and our method (with or without GW-WB).

As shown in Figure 11, we zoom in on the details of the image for comparison to intuitively demonstrate the model’s restoration effect on the white background, texture area, and highlights. Mamba U-Net can effectively suppress the blue color cast of white background images under the action of the GW-WB module, while U-Net has a green color cast and many noise points in complex texture areas. Mamba U-Net restores the clear edges and fine-grained textures of plants on the ground through skip connections and multi-scale feature fusion, while the comparison method produces blurred results due to the limitations of local convolution; in the highlighted area of the light source, our method avoids overexposure through the selective state propagation mechanism and restores the natural halo of the light source, while U-Net loses details or distorts colors.

As illustrated in Table 5, this experiment assesses the optimization effects of the GW-WB module on the standard test set, utilizing the pre-trained weights of the baseline model (RGB U-Net) derived from the original implementation in the literature [19]. It is important to note that the baseline model has undergone multiple iterations, which may introduce systemic errors when directly using the original pre-trained weights. Nonetheless, the experimental data indicate that introducing the GW-WB module improves the PSNR and SSIM of the reconstructed images by 0.22 dB and 0.004, respectively. Further analysis reveals that the impact of GW-WB on PSNR is primarily concentrated in color-sensitive areas, while its effect on the brightness component remains negligible. This suggests that GW-WB enhances the overall index mainly by correcting color distortions rather than contributing to structural reconstruction, aligning with its design objectives.

## 5. Conclusions

In this study, we utilized linear interpolation to transform the four-dimensional RGGB image into a three-dimensional RGB image. Following this reduction, we eliminated the black level and processed the image using the Mamba U-Net network to achieve the final result. The experimental outcomes demonstrate that our method surpasses other single-step end-to-end training approaches and certain multi-step methods in terms of data quality, achieving a numerical PSNR improvement of 0.06 dB and an SSIM [3] advantage of 0.003. Furthermore, while preserving acceptable imaging quality, our method nearly halves the training duration, resource consumption, and image generation time by capitalizing on the characteristics of the Mamba module and the efficiency of the single-step process.

This study still has some limitations, which provide clear directions for future research. First, although the hyperparameters currently used, such as training period, network depth, and learning rate, are based on existing benchmark methods to ensure reproducibility, the sensitivity of these parameters in different scenarios still needs further systematic validation. For example, adaptive tuning for different lighting conditions or sensor types may further enhance the robustness of the model. Future work will explore automation parameter optimization strategies to more comprehensively adapt to diverse extremely dark-light environment requirements. Second, the existing evaluation metrics mainly use PSNR and SSIM to focus on perceived quality, but in the future, more relevant evaluation metrics such as spectral angle mapper (SAM) can be used to demonstrate the effectiveness of our method. Finally, although Mamba U-Net has achieved a balance between efficiency and performance, recent research [44] on Mamba-like architectures [45,46,47] suggests that dynamic state space configuration may further optimize model performance, but it requires a trade-off in computational resource consumption. How to improve reconstruction quality without significantly increasing costs remains a key challenge in practical deployment.

## Figures and Tables

**Table 1:** Resultant file sizes for bilinear interpolation on four different types of image downscaling and comparison with the Python raw package method.

Type	Original RGGB File Size	Python Rawpy Package RGB File Size	Bilinear Interpolation RGB File Size
Black Background Object	23.57 MB	19.37 MB	1.21 MB
Indoor	23.5 MB	11.62 MB	0.41 MB
Object	23.5 MB	14.81 MB	0.88 MB
Outdoor	23.55 MB	15.63 MB	1.13 MB

**Table 2:** Performance comparison of interpolation methods on extreme dark-light RGGB images. Bilinear interpolation achieves near-ideal quality (SSIM > 0.97) with minimal resource consumption. “**↑**” stands for the higher data value, the better the performance.

Type	Python Rawpy Package PSNR↑/SSIM↑	Bilinear Interpolation PSNR↑/SSIM↑
Black Background Object	41.94/0.8805	45.70/0.9734
Indoor	44.47/0.9974	46.12/0.9993
Object	47.09/0.9943	48.93/0.9996
Outdoor	37.20/0.8758	48.93/0.9841

**Table 3:** Comparison among various methods for the SID [18] Sony dataset (image) using PSNR and SSIM metrics. “**↑**” stands for the higher data value, the better the performance.

Category	Method	PSNR↑	SSIM↑
Single-Stage	DID [21]	29.16	0.785
	SGN [22]	29.28	0.790
	LLPackNet [23]	27.83	0.755
	RRT [24]	28.66	0.790
	Self-Attention U-Net [6]	29.17	0.788
	Ours	29.34	0.793
Multi-Stage	EEMEFN [25]	29.60	0.795
	LDC [26]	29.56	0.799
	MCR [27]	29.65	0.797
	RRENet [28]	29.17	0.792
	DNF [29]	30.62	0.797
	Self-Attention + HDR [6]	30.78	0.799

**Table 4:** Single-stage training performance-resource efficiency comparison (SID dataset).

Type	Parameters	FLOPs
SID [18]	7.7 M	48.5 G
Single Self-Attention [6]	33.4 M	148.7 G
Proposed Mamba U-Net	2.3 M	20.9 G

**Table 5:** PSNR and SSIM results from standard U-Net and our method (with or without GW-WB). “**↑**” stands for the higher data value, the better the performance.

Type	PSNR↑	SSIM↑
Standard U-Net RGB [19]	26.96	0.694
Proposed without GW-WB	29.12	0.789
Proposed with GW-WB	29.34	0.793

## Data Availability

The datasets used and/or analyzed during the current study are available from the corresponding author upon reasonable request.
